# Oligomerising biomimetic UV filters for next-generation sunscreens

**DOI:** 10.1039/d6cp02283a

**Published:** 2026-09-24

**Authors:** Jack Dalton, Zofia Majewska, Michael Hymas, Eva Domínguez, Antonio Heredia, Vasilios G. Stavros

**Affiliations:** a School of Chemistry, University of Birmingham Edgbaston B15 2TT UK j.dalton@bham.ac.uk v.stavros@bham.ac.uk; b Departamento de Mejora Genética y Biotecnología, Instituto de Hortofruticultura Subtropical y Mediterránea “La Mayora”, Universidad de Málaga-Consejo Superior de Investigaciones Científicas, Estación Experimental La Mayora 29750 Málaga Spain; c Departamento de Biología Molecular y Bioquímica, Instituto de Hortofruticultura Subtropical y Mediterránea “La Mayora”, Universidad de Málaga-Consejo Superior de Investigaciones Científicas, Universidad de Málaga 29071 Málaga Spain heredia@uma.es

## Abstract

*para*-Coumaric acid (pCA), a naturally occurring ultraviolet (UV) filter, was oligomerised to form its trimer, 3pCA, to explore its potential application for sunscreen photoprotection. 3pCA's photophysical and photochemical properties following the absorption of solar radiation were investigated through steady-state and ultrafast transient absorption spectroscopic methods. The results presented here imply a network of three largely independent pCA-like chromophore units within the whole molecular structure, that each utilise the isomerisable C=C double bond to facilitate ultrafast non-radiative decay. Complementing these ideal photodynamical properties, the oligomerisation additionally reduces solubility in many common organic solvents, opening the door for enhanced photoprotection through UV light scattering from aggregates, and brings the molecular weight closer to the 500 Daltons threshold suggested to minimise transcutaneous penetration. Altogether, this study demonstrates oligomerisation as an auspicious design principle for next-generation sunscreens.

## Abstract 2

An oligomer of naturally occurring *para*-coumaric acid was found to behave as largely independent monomer-like units, utilising photoisomerisation for ultrafast non-radiative decay while displaying various ideal physical properties for sunscreens.

## Graphical abstract

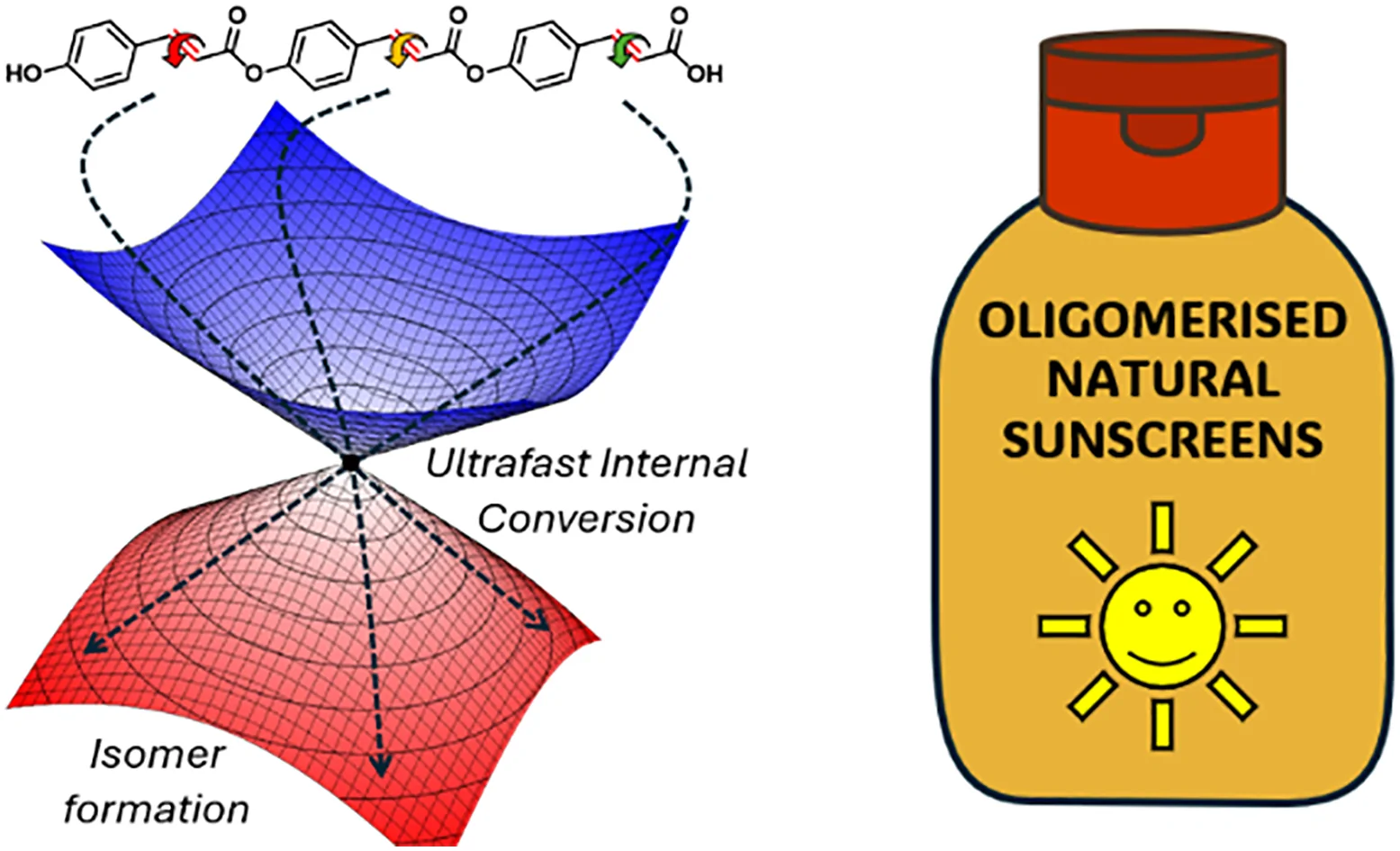

## Introduction

Plants are sensitive to excessive ultraviolet (UV) radiation and typically protect against its deleterious effects by the accumulation of phenolic ‘screening’ compounds, otherwise called ‘UV filters’, in their aerial parts.^1,2^ UV radiation is also well known to be hazardous to humans, causing melanomas and photoaging.^3–5^ Humans’ endogenous response (*i.e.*, melanin production, or tanning) is a delayed response and therefore fails to mitigate against initial UV-induced damage, necessitating the application of sunscreen formulations.^6,7^

The plant UV filter *para*-coumaric acid (pCA) is a secondary plant metabolite present in the cuticles of tomato fruits and is synthesised *via* the phenylpropanoid pathway.^8^ It has been investigated extensively and its photoprotection qualities at the outermost part of the plant epidermal cells (*i.e.*, the cuticle) are well understood.^9^pCA has a maximum absorption (*λ*_max_) in the UVB region (280–315 nm;^10^*λ*_max_ = 312 nm in ethanol) and, following UV excitation in solution, a *trans*–*cis* isomerisation coordinate facilitates non-radiative deactivation through a conical intersection (CI).^9,11,12^ At the CI, the dihedral angle of the acrylic double bond was found to be 90.3°, allowing subsequent vibrational cooling to either the *trans* or *cis* electronic ground state geometry.^12^

Artificial UV filter molecules used in sunscreen formulations continue to suffer from poor solar stability and undesired photochemistry under prolonged irradiation,^13–15^ as well as unsatisfactory UVA (315–400 nm)^10^ coverage^16^ and multifarious safety concerns.^17–20^ Phytochemical and biomimetic UV filters often exhibit less degradation and greater UVA attenuation than their artificial counterparts,^21^ however they are typically small molecules.^9^ As such, these molecules would not satisfy the so-called ‘500 Dalton rule’, which maintains that the chance of transcutaneous penetration is minimised if a dermally applied molecule's molecular weight is ≥500 Da.^22^

In this article, the oligomerisation of biomimetic UV filters as a design principle for next-generation sunscreens is studied, with the aim to overcome the abovementioned problems with UV filters. Here, a trimer of pCA, henceforth referred to as 3pCA, was synthesised and investigated through steady-state and ultrafast transient absorption spectroscopic methods. This oligomer (456.45 Da) is closer to meeting the ‘500 Dalton rule’ than its respective monomer (164.16 Da) and has the potential for a broadened and red-shifted absorbance compared to pCA. Steady-state measurements presented here confirm a broadened and red-shifted absorbance and suggest a network of three largely independent pCA-like chromophore units while, the transient absorption measurements reveal that the *trans*–*cis* isomerisation deactivation coordinate, observed previously for the monomer, is preserved in the oligomer to facilitate ultrafast non-radiative decay.

## Results and discussion

3pCA was found to be insoluble or sparingly soluble in many common organic solvents including methanol, water, cyclohexane, chloroform, diethyl ether, acetonitrile and tetrahydrofuran. 3pCA is soluble in dimethyl sulfoxide (DMSO), but unfortunately, DMSO's solvent window may promote unwanted photochemistry with solar radiation.^23^ To reproduce the polar-protic domains previously studied for pCA and to avoid potential solvent related photochemistry, all subsequent experiments were undertaken in methanol.^12^ The similar absorption profile of 3pCA in methanol and DMSO (see SI, Fig. S1) indicates no significant intermolecular electronic interactions from aggregates. To note, poor solubility in many organic solvents is not discouraging, but instead potentially favoured for UV light scattering, offering additional photoprotection similarly to inorganic filters (*e.g.*, ZnO and TiO_2_) and commercial nanoparticulate organic filters.^24–26^ One example being methylene bis-benzotriazolyl tetra-methylbutylphenol (MBBT) which has low solubility in water (<10^−6^%) and most cosmetic oils (<0.2%) and undergoes micronisation into nanoparticles of <200 nm to form a stable suspension in formulation.^24^ Preliminary dynamic light scattering measurements on 3pCA in methanol confirmed nanoparticles of approximately 100 nm (see SI, Fig. S2), which is optimal for UVA and UVB light scattering.^27–30^

Fig. 1 presents UV-visible absorption spectra of 3pCA in methanol before and at intervals throughout 24 h irradiation under a simulated solar spectrum (see SI, Fig. S3 for solar simulator's spectrum). Pre-irradiation 3pCA in methanol exhibits a *λ*_max_ between the UVA and UVB region at 315 nm, with a molar extinction coefficient (*ε*) of 42 700 M^−1^ cm^−1^ (see SI, Fig. S4). In comparison to the monomer,^12^3pCA's absorption is red-shifted, broader, and is over twice the peak absorbance (see SI, Fig. S5, for comparison), all of which are desirable for sunscreen applications.^31^ The dramatic increase in molar absorbance alongside a modest spectral red shift (5 nm) in absorbance of 3pCA compared to pCA suggests 3pCA behaves as three largely independent pCA-like chromophore units, without additional extended conjugation across the trimer. However, the deviation from the expected threefold increase in molar absorbance likely arises from non-equivalent absorption contributions of the pCA-like chromophore units, which naturally differ from pCA in the connecting groups such that there are three different pCA-like chromophore units within 3pCA. Time dependent density functional theory (TD-DFT) vertical excitation energy calculations were carried out on these different pCA-like chromophore units, confirming the non-equivalent absorption contributions with variations in both excitation energy and oscillator strengths to their first electronically excited singlet states (see SI, Fig. S6).

**Fig. 1:**
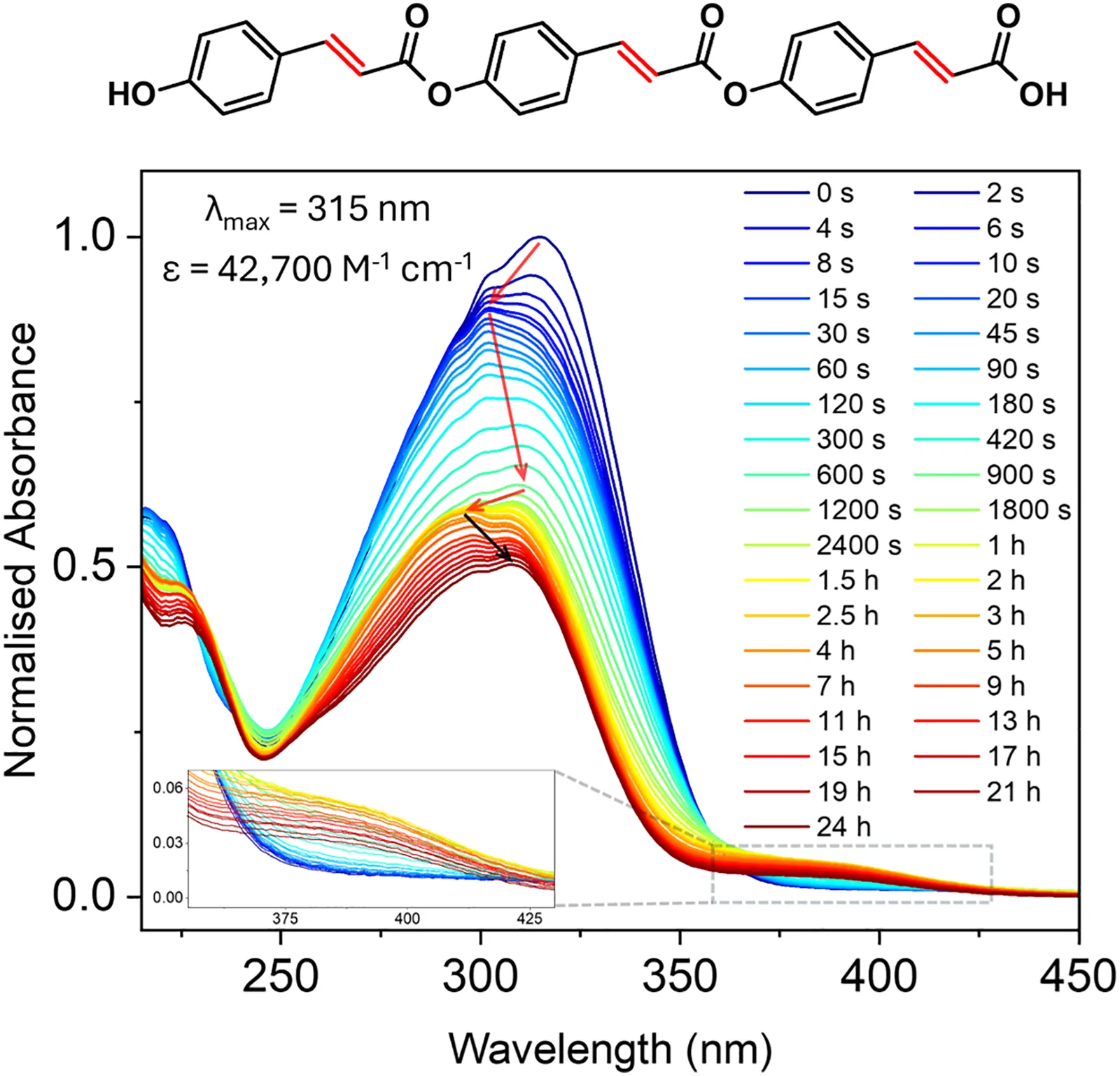
(top) Structure of 3pCA with isomerisable C=C bonds highlighted in red. (bottom) UV-visible absorption spectra of 3pCA at 10 µM in methanol before and during 24 h solar simulator irradiation. Red arrows highlight the multi-rate photostationary states reached from photoisomerisation while the black arrow highlights potential photodegradation.

Following solar irradiation, the absorption spectra display multiple spectral changes (predominantly highlighted by arrows in Fig. 1, guided by evolution associated spectra discussed below). The changes indicated by the red arrows are indicative of multiple photoisomerisation processes occurring and producing multiple photostationary states (PSS) (photoisomerisable C=C double bonds highlighted in Fig. 1), as repeatedly seen for other photoisomerisable biomimetic UV filters.^32–38^ The rate of individual PSS formation (at 10 µM following solar irradiation) can be obtained with a global sequential fitting model in the software package Glotaran to return quantitative insight and evolution associated spectra.^39^ The observed rate constant for reaching a PSS (*k*_PSS_; equal to the sum of the rate constants of *trans* to *cis*, *k*_*trans*→*cis*_, and *cis* to *trans*, *k*_*cis*→*trans*_, photoisomerisation) is dependent on the photon flux (which is constant), the absorption cross sections (*σ*; for each pCA-like unit in its *trans*, *σ*_*trans*_, and *cis*, *σ*_*cis*_, form) and the quantum yields for photoisomerisation (*ϕ*; for each pCA-like unit going from *trans* to *cis*, *ϕ*_*trans*→*cis*_, and *cis* to *trans*, *ϕ*_*cis*→*trans*_).^40^ Here, we observe four distinct rate constants (and therefore rates) of PSS formation: *k*_1_ = 1.60 × 10^−1^ s^−1^, *k*_2_ = 2.65 × 10^−3^ s^−1^, *k*_3_ = 4.56 × 10^−4^ s^−1^ and *k*_4_ = 1.23 × 10^−5^ s^−1^ (see SI, Fig. S7 for evolution associated spectra, fitting errors and residuals). The three fastest rates are assumed to account for the initial formation of *trans*–*cis* PSS of the three distinct pCA-like units, from comparison to the PSS formation timescales observed in similar photoisomerisable systems, which use an equivalent irradiance to what is used here.^12,33,41–44^ If we make the assumption that each pCA-like unit has a similar *σ*_*trans*_ (which is reasonable according to the vertical energy TD-DFT calculations presented in SI, Fig. S6) and that the *σ*_*trans*_/*σ*_*cis*_ ratios are equivalent between each unit, then the overall *ϕ* (*ϕ*_*trans*→*cis*_ + *ϕ*_*cis*→*trans*_) for each unit is proportional to *k*_PSS_. Under this assumption, the overall *ϕ* for each unit is drastically different due to the large differences in PSS formation rates. In reality, *σ*'s and *σ*_*trans*_/*σ*_*cis*_ ratios will be different between units, but it is unlikely to account for the observed 5.8- (*k*_2_/*k*_3_), 60- (*k*_1_/*k*_2_) and 350-fold (*k*_1_/*k*_3_) differences in PSS formation rates. The final, and very long, rate (*k*_4_) is assigned primarily to degradation (loss of molecular integrity) since loss of absorbance is observed across the UV spectrum, and PSS formation is not observed.

Within the first 2 hours of solar irradiation the area under the curve index (AUCI = 100% × AUC post-irradiation/AUC pre-irradiation; where AUC is short for the area under the curve) drops to 60% in the UVA and UVB region (SI, Fig. S8), primarily resulting from photoisomerisation and the formation of three PSS. A previously applied criterion for a photostable molecule (*i.e.*, a molecule whose molecular integrity is maintained despite irradiation) is an AUCI of >80% following 2 hours of irradiation;^14,45^ however, this criterion overlooks photochemistry which maintains molecular integrity but results in a loss of absorption, such as photoisomerisation. It is possible to overcome this criterion with 3pCA under the same conditions by starting with a mixture of photoisomers present at the third PSS formation point. Subsequent irradiation after the third PSS formation point results in minimal changes in UVA and UVB absorption such that a further 10% drop to an AUCI = 50% takes a total of 16.5 hours of solar simulator irradiation (SI, Fig. S8). Since the majority of UVA and UVB absorption loss results from photoisomerisation and not the loss of molecular integrity, one can conclude that 3pCA is photostable. NMR was attempted to assess the extent of photoisomerisation in deuterated methanol, however poor solubility precluded conclusive results.

Spurred by the observations of 3pCA under a solar spectrum, ultrafast transient electronic absorption spectroscopy was employed to uncover the ultrafast response of 3pCA to UV light. The collected transient absorption (TA) spectra and additional spectra at select time delays, following photoexcitation at the initial *λ*_max_ (315 nm), are presented in Fig. 2 and SI Fig. S9 respectively. Within the first 10 ps there are three main transient photophysical features for 3pCA: a negative feature around 325 nm, assigned to ground state bleach (GSB) from comparison with the ground state absorption spectrum (Fig. 1); a negative feature centred at 440 nm, assigned to stimulated emission (SE) from comparison with the emission spectrum (SI, Fig. S11); and two positive features concentrated at 360 nm and spanning 500–740 nm, which are assigned to excited state absorption (ESA) from the populated excited state to higher lying electronic states. Following decay of these features, a long-lived negative and positive feature at 335 and 375 nm, respectively, appear and remain beyond our maximum experimental time window (1.9 ns). These long-lived features show remarkable agreement with the difference in absorption between the pre- and post-irradiation with a solar simulator, presented in Fig. 3. This agreement confirms that the long-term photochemical changes (photoisomerisation) induced by UV radiation occur on the picosecond timescale. Three difference absorption spectra corresponding to selected post-irradiation times are displayed in Fig. 3 (specifically following 15, 600 and 7200 s irradiation), chosen to reflect timescales of the three distinct isomerisation processes of the pCA-like chromophore units. Since the longer post-irradiation times show better agreement with the TA spectrum at 1.9 ns, one can infer that multiple pCA-like chromophore units in the bulk solution are photoexcited in parallel and undergo non-radiative relaxation *via* photoisomerisation within the aforementioned decay of ESA, SE and GSB in the TA experiment (*i.e.*, on the picosecond timescale).

**Fig. 2:**
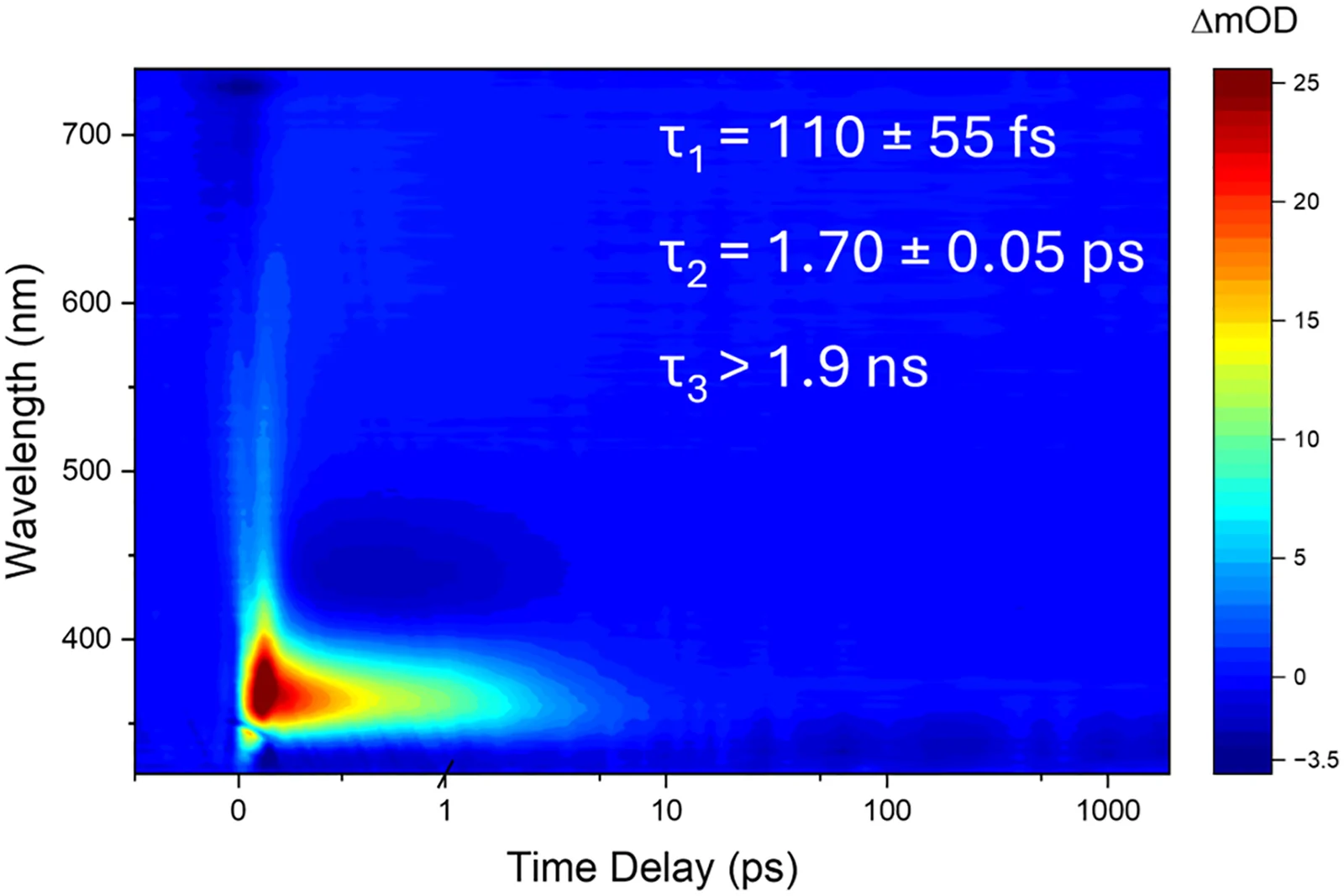
Transient electronic absorption spectra presented as a false colour heatmap for 3pCA at 65 µM in methanol following photoexcitation at 315 nm. The time delay is linear up to 1 ps and logarithmic from 1 to 1900 ps. Lifetimes extracted from global sequential fitting in the Glotaran software package are presented. The errors shown relate to half the instrument response (SI, Fig. S10) as it is greater than the fitting errors.

**Fig. 3:**
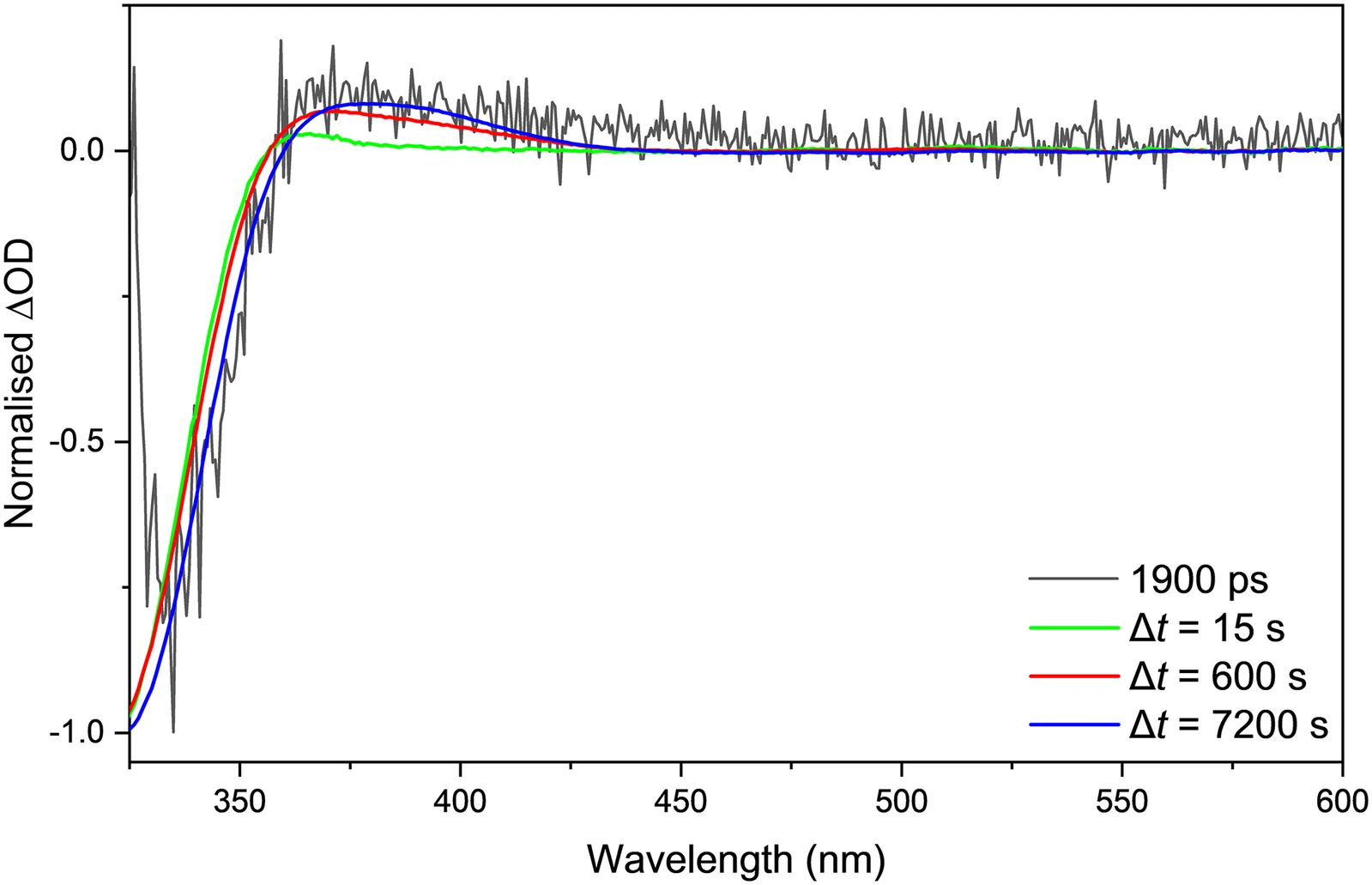
Comparison of the TA spectra of 3pCA in methanol at 1.9 ns following photoexcitation at 315 nm (black), and the difference absorption spectrum before and after 15 s (green; Δ*t* = 15 s), 600 s (red; Δ*t* = 600 s) and 7200 s (blue; Δ*t* = 7200 s) solar simulator irradiation (initial minus final).

For quantitative insight, global sequential fitting analysis of the TA data was undertaken in Glotaran,^39^ returning lifetimes of: *τ*_1_ = 110 ± 55 fs, *τ*_2_ = 1.70 ± 0.05 ps and *τ*_3_ > 1.9 ns. Evolution associated difference spectra (EADS) presented in SI Fig. S12 show *τ*_1_ to include mainly ESA with involvement of coherent artifacts from the solvent (see SI, Fig. S10), while *τ*_2_ includes contributions from GSB, ESA and SE; the SE implying population is still in the electronic excited state. Due to similarities in TA features and extracted lifetimes (with concurrent EADS) to that of pCA,^12^*τ*_1_ and *τ*_2_ are likewise assigned to: fast geometry relaxation within the initially populated electronic excited state towards a CI with the electronic ground state (*τ*_1_), followed by internal conversion through the CI and population of the electronic ground state (*τ*_2_). EADS for *τ*_3_ capture the long-lived positive and negative features from the formation of the isomer photoproduct indicating that photoisomerisation occurs within *τ*_1_ and *τ*_2_, facilitating non-radiative decay. Additionally, a negligible emission quantum yield was measured (<1%; data not included), consistent with the efficient non-radiative decay observed in the TA measurements.

Comparison with pCA^12^ demonstrates that oligomerisation results in minimal perturbations to the electronic excited states while preserving the non-radiative decay mechanism. This behaviour is highly desirable for sunscreen applications. Interestingly, it is somewhat unexpected, as the additional steric bulk associated with the neighbouring monomer units might be anticipated to restrict the nuclear motions involved in the photoisomerisation.

## Methods

### Synthesis

3pCA was synthesised in a multi-step synthesis starting from the commercially available pCA (1), outlined in Scheme 1. Protection of the phenol group using TBDMSCl, followed by activation of the carboxylic acid with SOCl_2_, afforded intermediate 2, which was then reacted with phenol (3) in acid media to give the esterified dimer 4. Subsequent deprotection and a new esterification with intermediate 2 provided compound 5. Finally, compound 3pCA was obtained by full deprotection of the protecting groups. The trimer was obtained with >95% purity and confirmed to be in the all *trans* form (see SI, Fig. S13). Synthesis of the pentamer was attemped, but failed due to solubility issues.

**Scheme 1:**
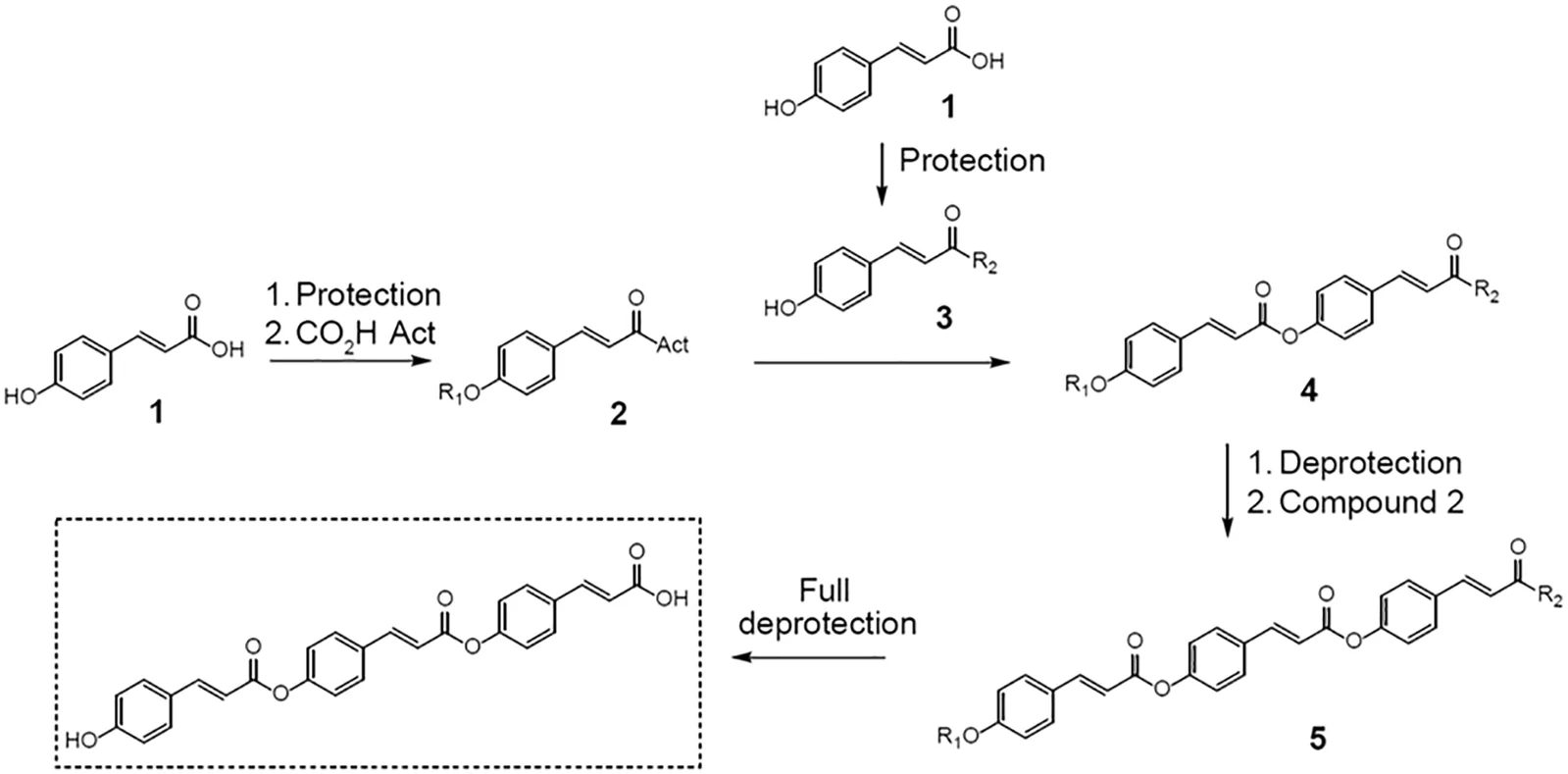
Synthesis of 3pCA.

### Steady-state spectroscopy

The absorption spectra of 3pCA in methanol were taken in a 1 cm path-length quartz cuvette using a Cary 60 spectrometer (Agilent Technologies). An Oriel LCS-100 solar simulator with an AM 1.5G spectral correction filter (see SI, Fig. S3, for spectrum) was used for the solar simulator irradiation measurements; the sample was positioned such that the irradiance was equivalent to one Sun, a unit of power flux which corresponds to the irradiance on the surface of the Earth on a clear summer day (∼1000 W m^−2^).

Photostationary state formation rates from steady-state irradiation absorption spectra were obtained from sequential global fitting 
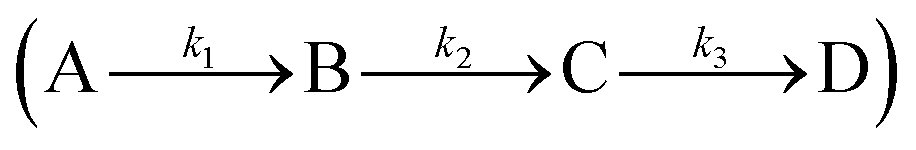
 in the software package Glotaran.^39^ The obtained rate constants correspond to evolution-associated spectra (SI, Fig. S7b), representing the spectral features that evolve at that rate. The quality of the fit can be assessed through inspection of the fitting residuals (SI, Fig. S7c).

The emission spectrum of 3pCA in methanol was taken using a Cary Eclipse fluorescence spectrometer (Agilent Technologies) with an excitation wavelength of 315 nm. A 1 cm path length quartz cuvette was used, and the solution concentration was made up to a maximum absorbance of 0.15 to reduce inner filter effects. Emission was collected at 90° to the sample excitation geometry.

### Ultrafast transient electronic absorption spectroscopy

A detailed overview of the transient electronic absorption spectroscopy experimental setup has been outlined elsewhere;^46^ what follows is a brief overview, including details specific to this experiment. A Ti-sapphire regenerative amplifier laser system (Spectra-Physics, Ascend 60 pumped Spitfire Ace) seeded by a Mai Tai (Spectra-Physics) produced the fundamental 800 nm pulsed beam (7 W, 1 kHz, ∼35 fs). ∼1 W seeded an optical parametric amplifier (light conversion, TOPAS-C) to produce the 315 nm pump beam. Every other pump pulse was blocked, and the beam was attenuated to ∼250 µW before being focused behind the sample to give a beam diameter of ∼300–500 µm at the sample. Another ∼0.15 W of the fundamental 800 nm beam was optically delayed producing pump–probe time delays ranging from −1 ps to 1.9 ns, before the polarisation was adjusted to magic angle (54.7°) relative to the pump and subsequently focused into a CaF_2_ window to generate the 320–740 nm white light supercontinuum probe. The probe was focused into the sample, overlapping with the pump, and detected by a fibre-coupled spectrometer (Avantes Ltd, dual channel Avaspec-Fast, ULS1650F-2-USB2). The change in optical density (ΔOD) of the probe between when the pump is ‘on’ or ‘off’ is calculated in a home built LabView program.

At the sample interaction region, a 65 µM solution of 3pCA in methanol was recirculated from a 25 mL reservoir through a flow cell (Demountable Liquid Cell, Harrick Scientific Products Inc.) using a peristaltic pump (Masterflex® MFLX07528-20-UK). The flow cell consisted of two 25 mm CaF_2_ windows (thickness: 1 mm front, 2 mm back) separated by 950 µm poly(tetrafluoroethylene) spacers, which defined the optical path length of the sample.

Quantitative information from the collected transient absorption spectra was obtained through sequential global fitting in the software package Glotaran.^39^ The model includes multiple sequential exponential decays convoluted with a Gaussian to model the instrument response (IR). The resulting lifetimes are linked to evolution-associated difference spectra (EADS) that represent the evolving spectral features relating to each lifetime extracted (SI, Fig. S12a). The quality of the fit can be assessed through the fitting residuals (SI, Fig. S12b). The IR for the experiment (110 fs) was estimated from a Gaussian fit of the kinetic trace at 360 nm from a solvent (methanol) scan under the same experimental conditions outlined above (SI, Fig. S10). To aesthetically improve the collected TA data, heatmaps were chirp-corrected using the software package KOALA, which utilised a third-order polynomial to model the dispersion.^47^

### Computational methods

Conformational sampling was performed using the conformer–rotamer ensemble sampling tool (CREST, V. 2.12)^48–50^ at the GFN2-xTB level of theory.^51^ The resulting structures were assessed for energy and conformational differences, including variations in dihedral angles and functional group positions, and representative conformers showing the greatest structural differences were selected for further calculations, within the limit threshold of 1 kcal mol^−1^. These structures were selected for further calculations. Ground state geometry optimisations and vertical excitation calculations were subsequently performed using DFT and TD-DFT, respectively, with the B3LYP functional, def2-TZVP basis set, and D3 dispersion correction. This method was chosen to reproduce the calculations previously performed on the pCA monomer subunit.^12^ Solvent effects were considered using CPCM model with methanol as an implicit solvent.^52,53^

## Conclusions

Oligomerisation of *para*-coumaric acid (pCA) to form its trimer (3pCA) retains the efficient ultrafast non-radiative decay mechanism of the monomer while introducing characteristics desirable for sunscreen applications. The increased molecular structure may reduce skin penetration, and the low solubility could confer additional UV protection through light scattering. Following UV excitation, 3pCA behaves as three largely independent pCA-like chromophores that dissipate absorbed energy (as heat) *via* a *trans*–*cis* photoisomerisation, resulting in minimal molecular photodegradation. Overall, these results establish oligomerisation as an effective biomimetic UV filter design strategy and highlights its potential applicability to a wider range of photoisomerisable natural-product-inspired chromophores including, but not limited to, sinapates, flavonoids and urocanates. Furthermore, we believe a systematic comparison of the monomer with subsequent addition of single monomer units would be of great value to further our understanding of the photophysical properties of oligomerised biomimetic UV filters.

## Conflicts of interest

There are no conflicts to declare.

## Supplementary Material

CP-028-D6CP02283A-s001

## Data Availability

The data supporting this article have been including as part of the supplementary information (SI). Supplementary information is available. See DOI: https://doi.org/10.1039/d6cp02283a. Raw data is available upon reasonable request from the corresponding authors.

## References

[cit1] Nunes A. R., Vieira Í. G. P., Queiroz D. B., Leal A. L. A. B., Maia Morais S., Muniz D. F., Calixto-Junior J. T., Coutinho H. D. M. (2018). Use of Flavonoids and Cinnamates, the Main Photoprotectors with Natural Origin. Adv. Pharmacol. Pharm. Sci..

[cit2] Matthew Robson T., Aphalo P. J., Banaś A. K., Barnes P. W., Brelsford C. C., Jenkins G. I., Kotilainen T. K., Łabuz J., Martínez-Abaigar J., Morales L. O., Neugart S., Pieristè M., Rai N., Vandenbussche F., Jansen M. A. K. (2019). A Perspective on Ecologically Relevant Plant-UV Research and Its Practical Application. Photochem. Photobiol. Sci..

[cit3] Fisher G. J., Wang Z., Datta S. C., Varani J., Kang S., Voorhees J. J. (1997). Pathophysiology of Premature Skin Aging Induced by Ultraviolet Light. N. Engl. J. Med..

[cit4] de Gruijl F. R. (1999). Skin Cancer and Solar UV Radiation. Eur. J. Cancer.

[cit5] Narayanan D. L., Saladi R. N., Fox J. L. (2010). Review: Ultraviolet Radiation and Skin Cancer. Int. J. Dermatol..

[cit6] Lautenschlager S., Wulf H. C., Pittelkow M. R. (2007). Photoprotection. Lancet.

[cit7] Eller M. S., Gilchrest B. A. (2000). Tanning as Part of the Eukaryotic SOS Response. Pigm. Cell Res..

[cit8] Agati G., Brunetti C., Di Ferdinando M., Ferrini F., Pollastri S., Tattini M. (2013). Functional Roles of Flavonoids in Photoprotection: New Evidence, Lessons from the Past. Plant Physiol. Biochem..

[cit9] González Moreno A., de Cózar A., Prieto P., Domínguez E., Heredia A. (2022). Radiationless Mechanism of UV Deactivation by Cuticle Phenolics in Plants. Nat. Commun..

[cit10] Coblentz W. W. (1932). The Copenhagen Meeting of the Second International Congress on Light. Science.

[cit11] Groenhof G., Lensink M. F., Berendsen H. J. C., Mark A. E. (2002). Signal Transduction in the Photoactive Yellow Protein. II. Proton Transfer Initiates Conformational Changes. Proteins: Struct., Funct., Bioinf..

[cit12] González Moreno A., Woolley J. M., Domínguez E., de Cózar A., Heredia A., Stavros V. G. (2023). Synergic Photoprotection of Phenolic Compounds Present in Tomato Fruit Cuticle: A Spectroscopic Investigation in Solution. Phys. Chem. Chem. Phys..

[cit13] Mturi G. J., Martincigh B. S. (2008). Photostability of the Sunscreening Agent 4-*tert*-Butyl-4′-Methoxydibenzoylmethane (Avobenzone) in Solvents of Different Polarity and Proticity. J. Photochem. Photobiol., A.

[cit14] Hojerová J., Medovcíková A., Mikula M. (2011). Photoprotective Efficacy and Photostability of Fifteen Sunscreen Products Having the Same Label SPF Subjected to Natural Sunlight. Int. J. Pharm..

[cit15] Holt E. L., Rodrigues N. D. N., Cebrián J., Stavros V. G. (2021). Determining the Photostability of Avobenzone in Sunscreen Formulation Models Using Ultrafast Spectroscopy. Phys. Chem. Chem. Phys..

[cit16] Fourtanier A., Moyala D., Seite S. (2012). UVA Filters in Sun-Protection Products: Regulatory and Biological Aspects. Photochem. Photobiol. Sci..

[cit17] Suh S., Pham C., Smith J., Mesinkovska N. A. (2020). The Banned Sunscreen Ingredients and Their Impact on Human Health: A Systematic Review. Int. J. Dermatol..

[cit18] Matta M. K., Zusterzeel R., Pilli N. R., Patel V., Volpe D. A., Florian J., Oh L., Bashaw E., Zineh I., Sanabria C., Kemp S., Godfrey A., Adah S., Coelho S., Wang J., Furlong L.-A., Ganley C., Michele T., Strauss D. G. (2019). Effect of Sunscreen Application Under Maximal Use Conditions on Plasma Concentration of Sunscreen Active Ingredients: A Randomized Clinical Trial. JAMA.

[cit19] Maipas S., Nicolopoulou-Stamati P. (2015). Sun Lotion Chemicals as Endocrine Disruptors. Hormones.

[cit20] Sharma A., Bányiová K., Babica P., El Yamani N., Collins A. R., Čupr P. (2017). Different DNA Damage Response of Cis and Trans Isomers of Commonly Used UV Filter after the Exposure on Adult Human Liver Stem Cells and Human Lymphoblastoid Cells. Sci. Total Environ..

[cit21] Baker L. A., Horbury M. D., Greenough S. E., Allais F., Walsh P. S., Habershon S., Stavros V. G. (2016). Ultrafast Photoprotecting Sunscreens in Natural Plants. J. Phys. Chem. Lett..

[cit22] Bos J. D., Meinardi M. M. H. M. (2000). The 500 Dalton Rule for the Skin Penetration of Chemical Compounds and Drugs. Exp. Dermatol..

[cit23] Wrathall N. J., Eller J., Barker J. E., Rodrigues N. D. N., O’Reilly R. K., Hine N. D. M., Stavros V. G. (2025). Towards Elucidating the Solar Instability of the Anti-Fungal Food Preservative Natamycin: Insights from Spectroscopy. Phys. Chem. Chem. Phys..

[cit24] Herzog B., Hüglin D., Borsos E., Stehlin A., Luther H. (2004). New UV Absorbers for Cosmetic Sunscreens – A Breakthrough for the Photoprotection of Human Skin. Chimia.

[cit25] Stiefel C., Schwack W. (2015). Photoprotection in Changing Times – UV Filter Efficacy and Safety, Sensitization Processes and Regulatory Aspects. Int. J. Cosmet. Sci..

[cit26] Sohn M., Staudt A., Quass K., Herzog B. (2025). Scattering as Part of the Protection Afforded by Nanoparticulate Filters – An Example with Tris-Biphenyl Triazine. Int. J. Cosmet. Sci..

[cit27] Herzog B., Bressel L., Pulbere S., Reich O. (2024). Monte Carlo Simulations of Light Transport in Sunscreen Formulations. Photochem. Photobiol. Sci..

[cit28] Egerton T. A., Tooley I. R. (2012). UV Absorption and Scattering Properties of Inorganic-Based Sunscreens. Int. J. Cosmet. Sci..

[cit29] Herzog B., Quass K., Schmidt E., Müller S., Luther H. (2004). Physical Properties of Organic Particulate UV Absorbers Used in Sunscreens: II. UV-Attenuating Efficiency as Function of Particle Size. J. Colloid Interface Sci..

[cit30] Herzog B. (2026). Efficacy of UV-Attenuation by Organic Particulate UV Filters in Sunscreens Depends on Particle Size. JCIS Open.

[cit31] Baker L. A., Marchetti B., Karsili T. N. V., Stavros V. G., Ashfold M. N. R. (2017). Photoprotection: Extending Lessons Learned from Studying Natural Sunscreens to the Design of Artificial Sunscreen Constituents. Chem. Soc. Rev..

[cit32] Challice J. S., Williams A. H. (1966). Paper Chromatographic Separation and Behavior of the Cis- and Trans-Isomers of Cinnamic Acid Derivatives. J. Chromatogr. A.

[cit33] Woolley J. M., Peters J. S., Turner M. A. P., Clarkson G. J., Horbury M. D., Stavros V. G. (2019). The Role of Symmetric Functionalisation on Photoisomerisation of a UV Commercial Chemical Filter. Phys. Chem. Chem. Phys..

[cit34] Fan J., Wooley J. M., Sanders H., Stavros V. G., Buma W. J. (2024). Urocanic Acid as a Novel Scaffold for Next-Gen Nature-Inspired Sunscreens: II. Time-Resolved Spectroscopy under Solution Conditions. Phys. Chem. Chem. Phys..

[cit35] Luo J., Liu Y., Yang S., Flourat A. L., Allais F., Han K. (2017). Ultrafast Barrierless Photoisomerization and Strong Ultraviolet Absorption of Photoproducts in Plant Sunscreens. J. Phys. Chem. Lett..

[cit36] Baker L. A., Staniforth M., Flourat A. L., Allais F., Stavros V. G. (2018). Gas-Solution Phase Transient Absorption Study of the Plant Sunscreen Derivative Methyl Sinapate. ChemPhotoChem.

[cit37] Horbury M. D., Baker L. A., Quan W.-D., Greenough S. E., Stavros V. G. (2016). Photodynamics of Potent Antioxidants: Ferulic and Caffeic Acids. Phys. Chem. Chem. Phys..

[cit38] Horbury M. D., Baker L. A., Rodrigues N. D. N., Quan W.-D., Stavros V. G. (2017). Photoisomerization of Ethyl Ferulate: A Solution Phase Transient Absorption Study. Chem. Phys. Lett..

[cit39] Snellenburg J. J., Laptenok S., Seger R., Mullen K. M., van Stokkum I. H. M. (2012). Glotaran: A Java-Based Graphical User Interface for the R Package TIMP. J. Stat. Software.

[cit40] Stranius K., Börjesson K. (2017). Determining the Photoisomerization Quantum Yield of Photoswitchable Molecules in Solution and in the Solid State. Sci. Rep..

[cit41] Hymas M., Dalton J., Rioux B., Flourat A. L., Allais F., Omidyan R., Stavros V. G. (2025). Environment-Dependent Ultrafast Photodynamics of Aurone Derivatives for the Photoprotection of Agrochemicals. ChemPhotoChem.

[cit42] Horbury M. D., Turner M. A. P., Peters J. S., Mention M., Flourat A. L., Hine N. D. M., Allais F., Stavros V. G. (2020). Exploring the Photochemistry of an Ethyl Sinapate Dimer: An Attempt Toward a Better Ultraviolet Filter. Front. Chem..

[cit43] Hymas M., Casademont-Reig I., Poigny S., Stavros V. G. (2023). Characteristic Photoprotective Molecules from the Sphagnum World: A Solution-Phase Ultrafast Study of Sphagnic Acid. Molecules.

[cit44] Abiola T. T., Toldo J. M., do Casal M. T., Flourat A. L., Rioux B., Woolley J. M., Murdock D., Allais F., Barbatti M., Stavros V. G. (2022). Direct Structural Observation of Ultrafast Photoisomerization Dynamics in Sinapate Esters. Commun. Chem..

[cit45] Gonzalez H., Tarras-Wahlberg N., Strömdahl B., Juzeniene A., Moan J., Larkö O., Rosén A., Wennberg A.-M. (2007). Photostability of Commercial Sunscreens upon Sun Exposure and Irradiation by Ultraviolet Lamps. BMC Dermatol..

[cit46] Majewska Z., Eller J., Pan K., Dalton J., Herzog B., Hine N. D. M., Stavros V. G. (2026). Unraveling Solvent-Independent Excited State Proton Transfer Dynamics in Sterically Substituted Photoactive Systems. J. Phys. Chem. Lett..

[cit47] Grubb M. P., Orr-Ewing A. J., Ashfold M. N. R. (2014). KOALA: A Program for the Processing and Decomposition of Transient Spectra. Rev. Sci. Instrum..

[cit48] Grimme S. (2019). Exploration of Chemical Compound, Conformer, and Reaction Space with Meta-Dynamics Simulations Based on Tight-Binding Quantum Chemical Calculations. J. Chem. Theory Comput..

[cit49] Pracht P., Bohle F., Grimme S. (2020). Automated Exploration of the Low-Energy Chemical Space with Fast Quantum Chemical Methods. Phys. Chem. Chem. Phys..

[cit50] Pracht P., Grimme S., Bannwarth C., Bohle F., Ehlert S., Feldmann G., Gorges J., Müller M., Neudecker T., Plett C., Spicher S., Steinbach P., Wesołowski P. A., Zeller F. (2024). CREST—A Program for the Exploration of Low-Energy Molecular Chemical Space. J. Chem. Phys..

[cit51] Bannwarth C., Ehlert S., Grimme S. (2019). GFN2-XTB–An Accurate and Broadly Parametrized Self-Consistent Tight-Binding Quantum Chemical Method with Multipole Electrostatics and Density-Dependent Dispersion Contributions. J. Chem. Theory Comput..

[cit52] Barone V., Cossi M. (1998). Quantum Calculation of Molecular Energies and Energy Gradients in Solution by a Conductor Solvent Model. J. Phys. Chem. A.

[cit53] Cossi M., Rega N., Scalmani G., Barone V. (2003). Energies, Structures, and Electronic Properties of Molecules in Solution with the C-PCM Solvation Model. J. Comput. Chem..

