# Supplementary material for: Oligomerising biomimetic UV filters for next-generation sunscreens
Source: Phys Chem Chem Phys. 2026 Sep 24;28(40):24988–94. doi: 10.1039/d6cp02283a (PMC13626188; doi:10.1039/d6cp02283a)
Supplement: Supplementary file 1 [file CP-028-D6CP02283A-s001.pdf]

Supplementary Information (SI) for Physical Chemistry Chemical Physics.
This journal is © the Owner Societies 2026

Supplementary Information: Oligomerising Biomimetic UV Filters for Next-Generation Sunscreens

Jack Dalton,*[a] Zofia Majewska,[a] Michael Hymas,[a] Eva Domínguez,[b] Antonio Heredia*[c] and Vasilios G. Stavros*[a]

[a] School of Chemistry, University of Birmingham, Edgbaston, B15 2TT, United Kingdom.

[b] Departamento de Mejora Genética y Biotecnología, Instituto de Hortofruticultura Subtropical y Mediterránea "La Mayora", Universidad de Málaga-Consejo Superior de Investigaciones Científicas, Estación Experimental La Mayora, 29750 Málaga, Spain.

[c] Departamento de Biología Molecular y Bioquímica, Instituto de Hortofruticultura Subtropical y Mediterránea "La Mayora", Universidad de Málaga-Consejo Superior de Investigaciones Científicas, Universidad de Málaga, 29071 Málaga, Spain.

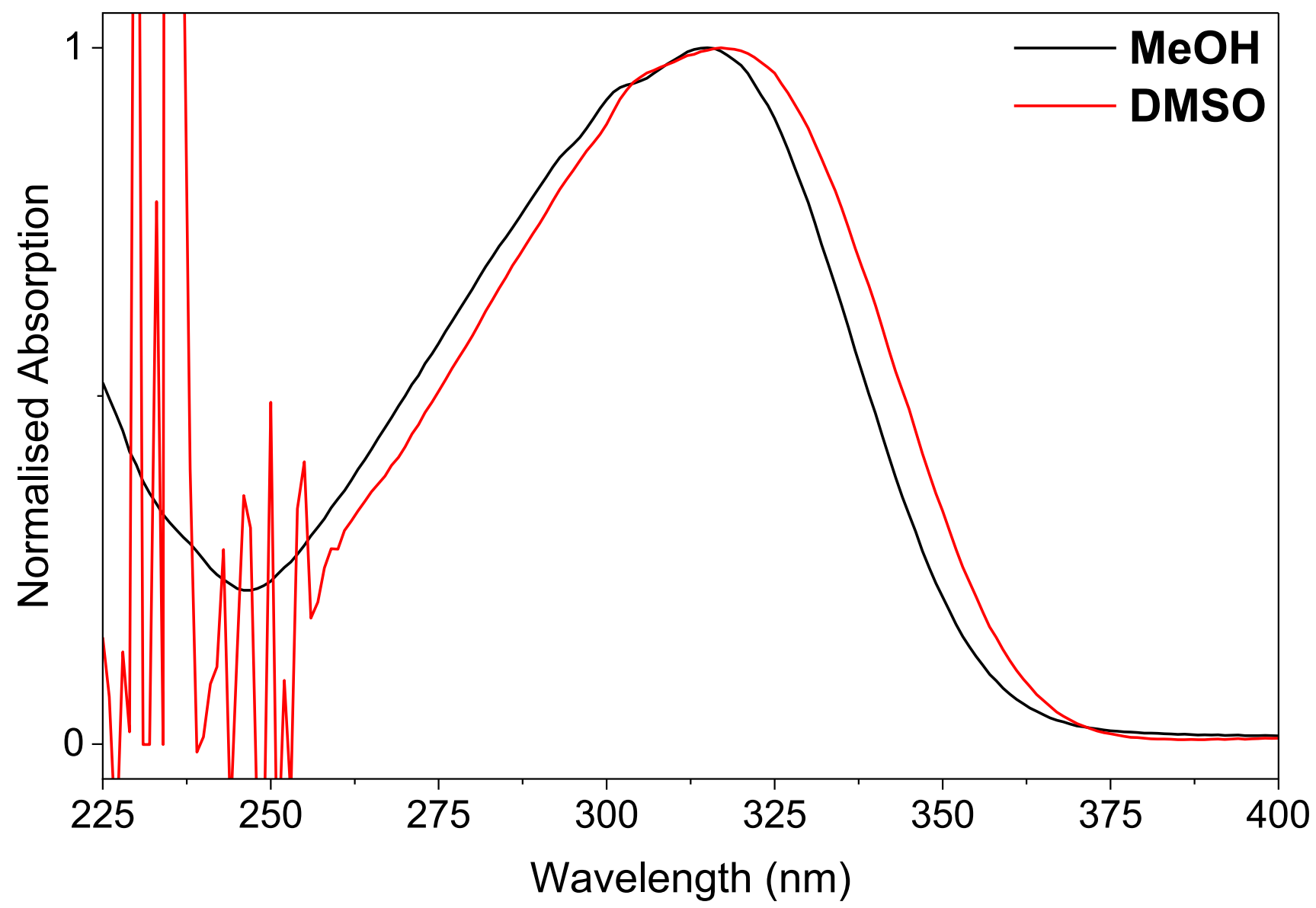

Figure S1 Normalised UV absorbance spectra for **3pCA** in methanol (MeOH, black line) and in dimethyl sulfoxide (DMSO, red line). The large spikes for the DMSO spectrum result from reaching the upper limit of quantification for the spectrometer due to DMSO's solvent window.

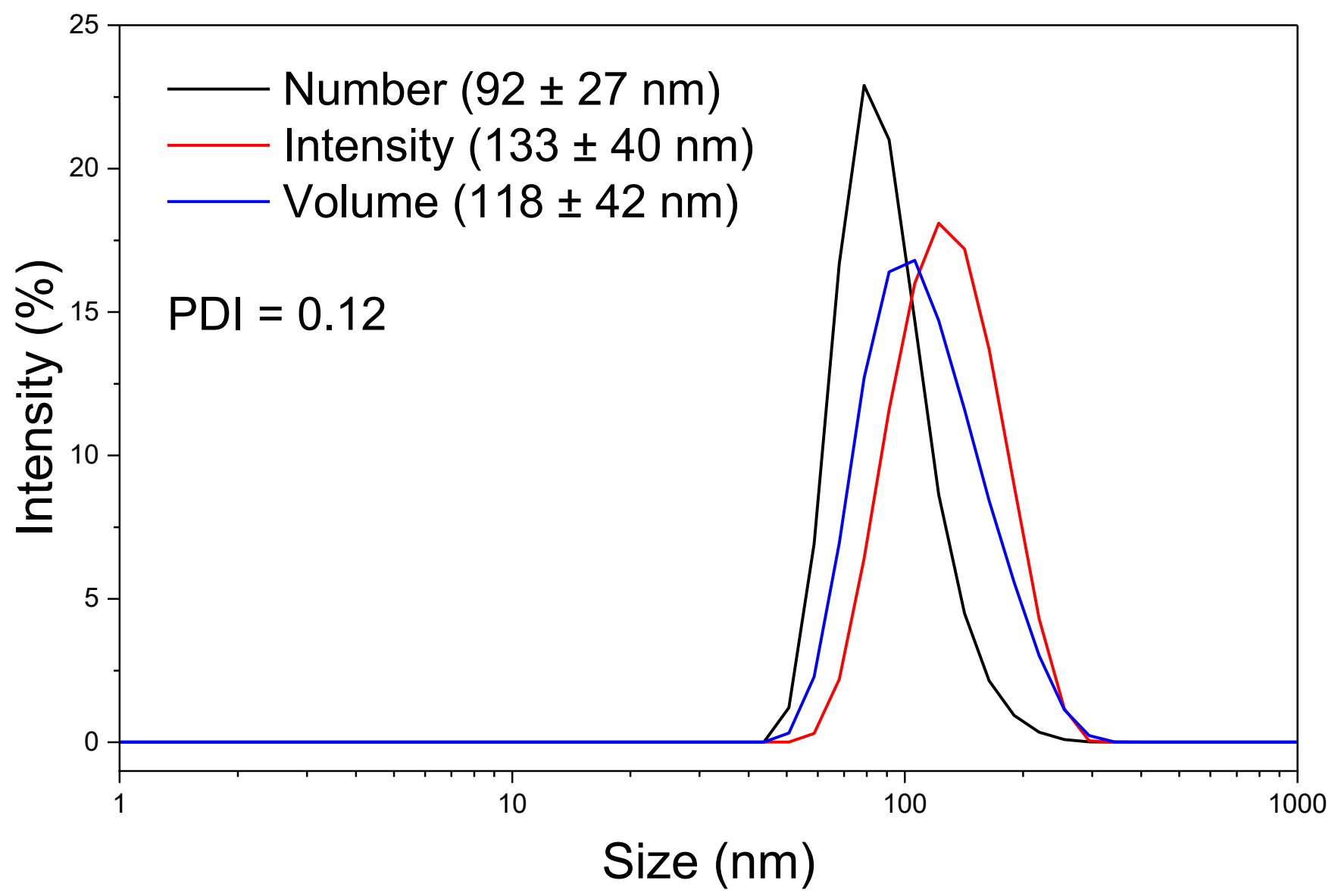

Figure S2 Dynamic light scattering measurement of **3pCA** in methanol to determine particle size. The solution (65 µM) was first sonicated and filtered through a polyethersulfone membrane 450 nm filter to remove very large particles (potentially dust or large aggregates) that caused inconsistent measurements. Black, red and blue lines display the number, intensity and volume distribution of the particles, respectively, with the averages and standard deviations displayed in the legend parentheses. The polydispersity index (PDI) is 0.12, indicating a near monodisperse range of particles sizes.

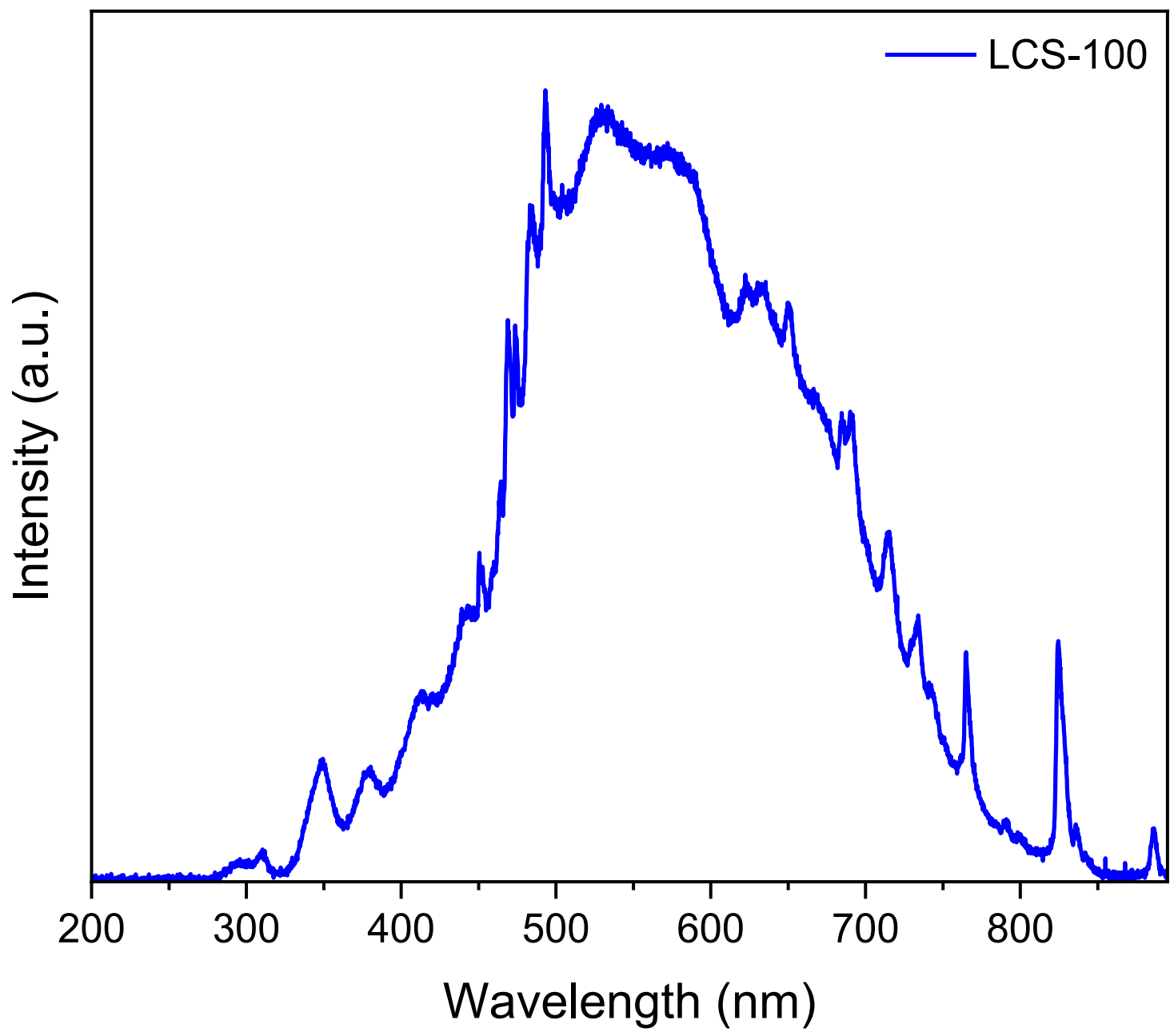

Figure S3 Spectral intensity of Oriel LCS-100 solar simulator with an AM 1.5G spectral correction filter used for irradiation measurements in Figure 1 of the main manuscript.

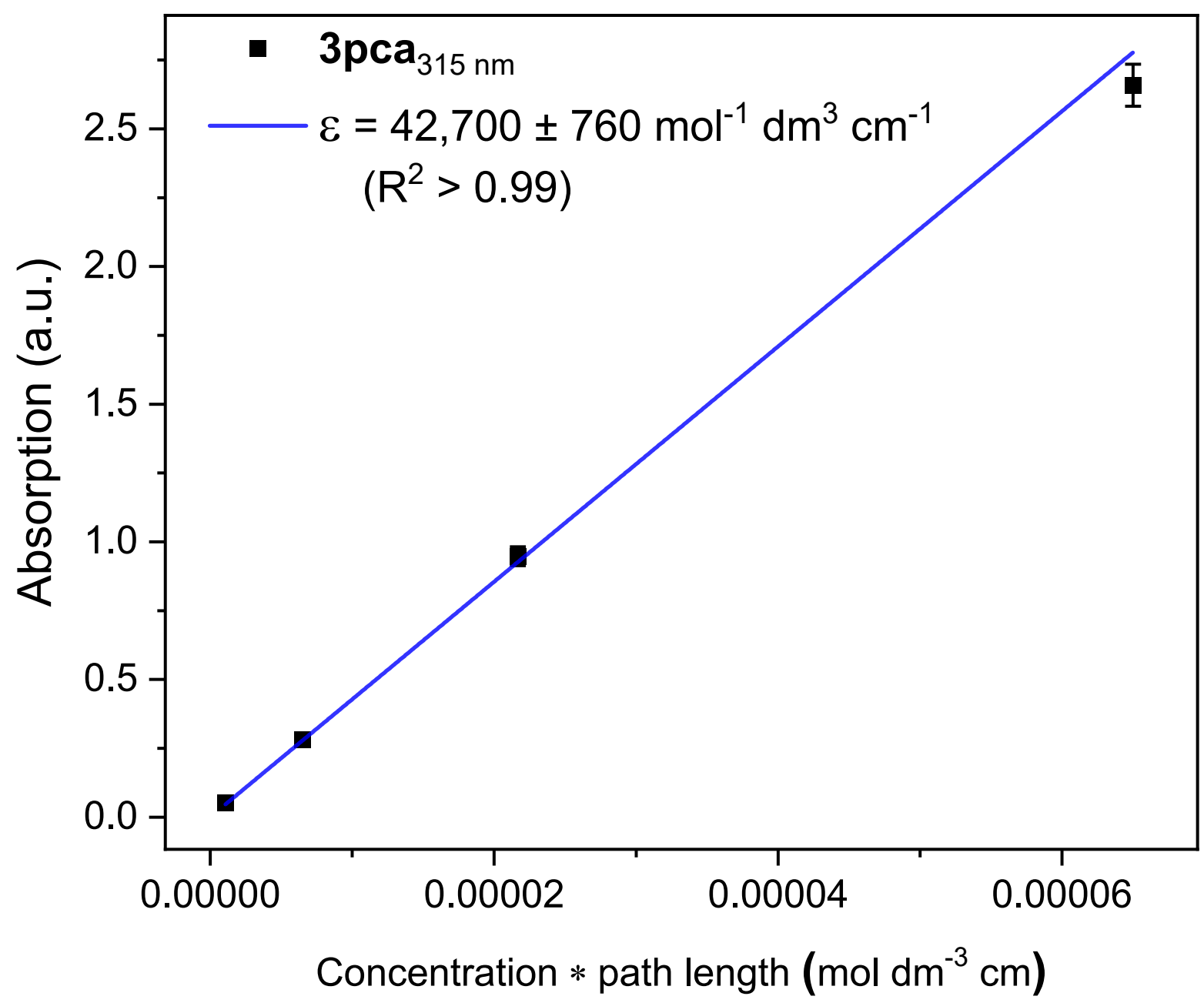

Figure S4 Absorption of **3pCA** in methanol at 315 nm against concentration of **3pCA** multiplied by the path length (1 cm). The gradient of the fitted line (blue) returned the molar extinction coefficient (ε) of 42,700 ± 760 mol^{-1} dm^3 cm^{-1}.

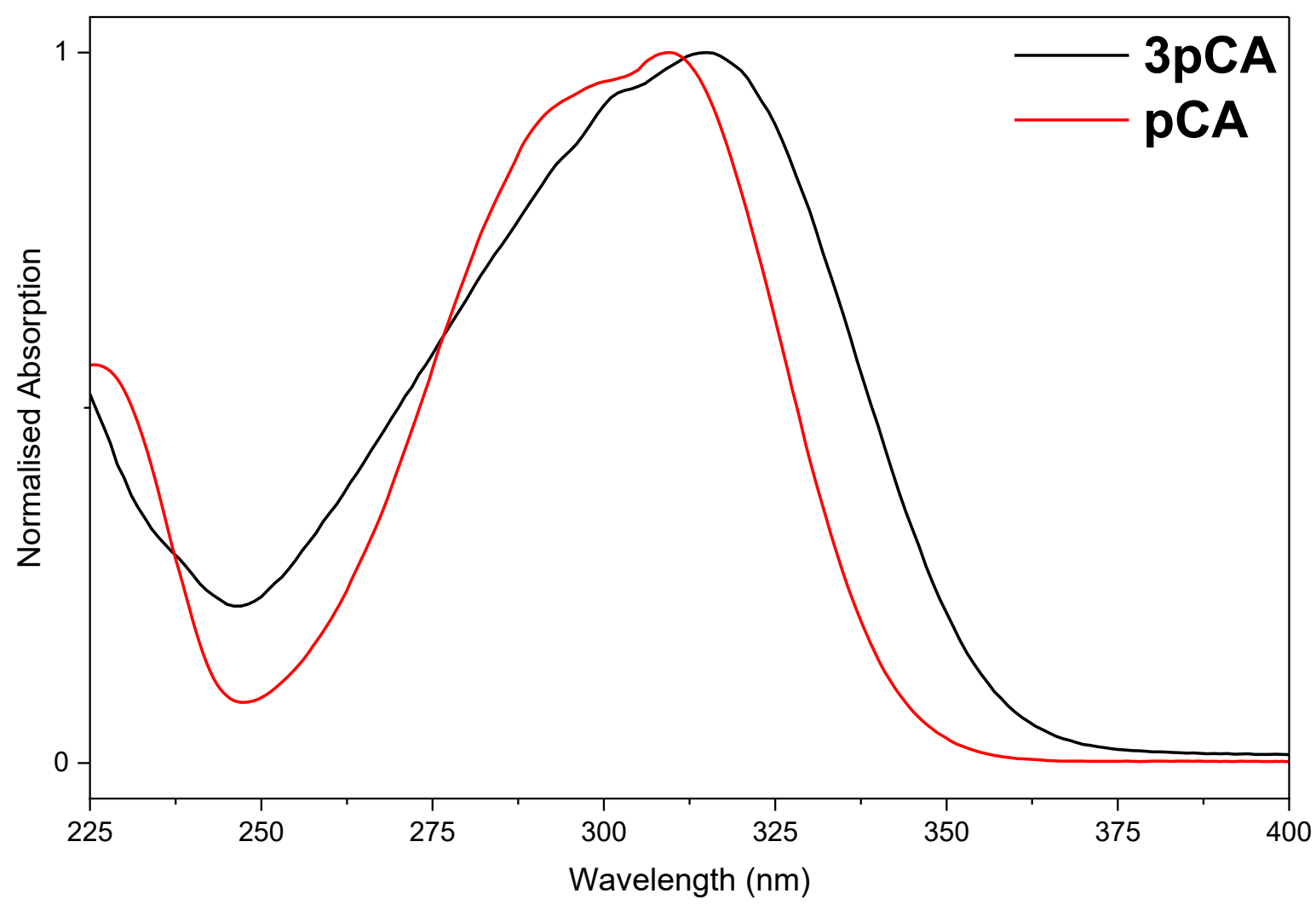

Figure S5 Absorption spectrum of **3pCA** (black line) and **pCA** (red line) in methanol.

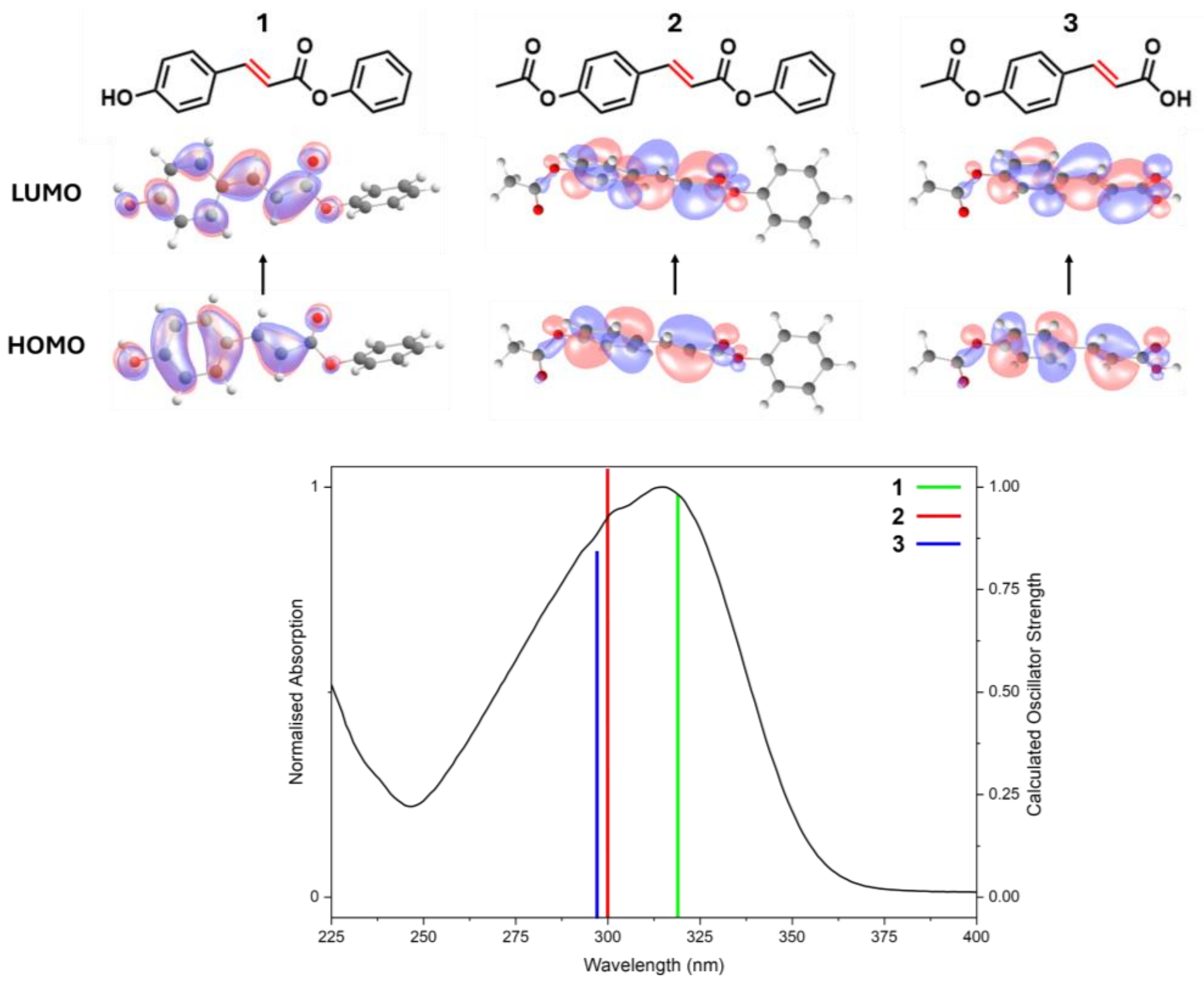

Figure S6 Time dependent density functional theory (TD-DFT) vertical excitation energy calculations with the B3LYP functional, def2-TZVP basis set and D3 dispersion correction in implicit methanol. Three **pCA**-like chromophore units from **3pCA** were selected, labelled **1**, **2** and **3** with the molecular structures presented at the top. Below the molecular structures presents the highest occupied molecular orbital (HOMO) and lowest unoccupied molecular orbital (LUMO) corresponding to the lowest energy singlet electronic excited state for each **pCA**-like chromophore unit (LUMO←HOMO; S_1). The wavelength and calculated oscillator strengths for the LUMO←HOMO transitions are presented in the bottom graph for **1** (green line), **2** (red line), and **3** (blue line), overlayed on the normalised absorption spectrum of **3pCA** in methanol (black line).

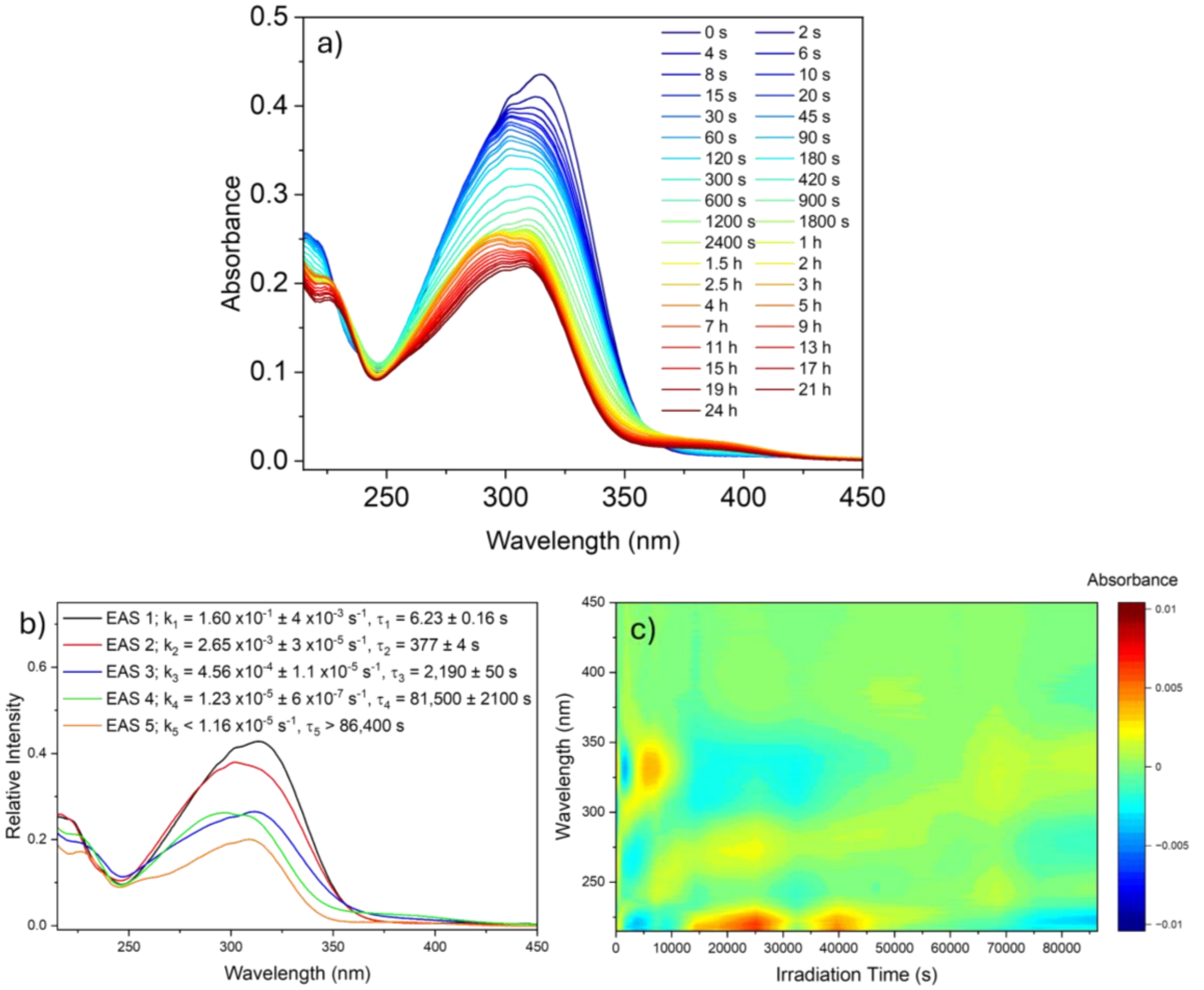

Figure S7 a) UV-visible absorption spectra of **3pCA** at 10 µM in methanol before and during 24 h solar simulator irradiation (path length of 1 cm; unnormalised data from Figure 1 of the main text). b) Evolution associated spectra (EAS) and the corresponding rate constants (k_x) and lifetimes (τ_x) obtained from global sequential fitting in the software package Glotaran. c) False colour heatmap of the fitting residuals.

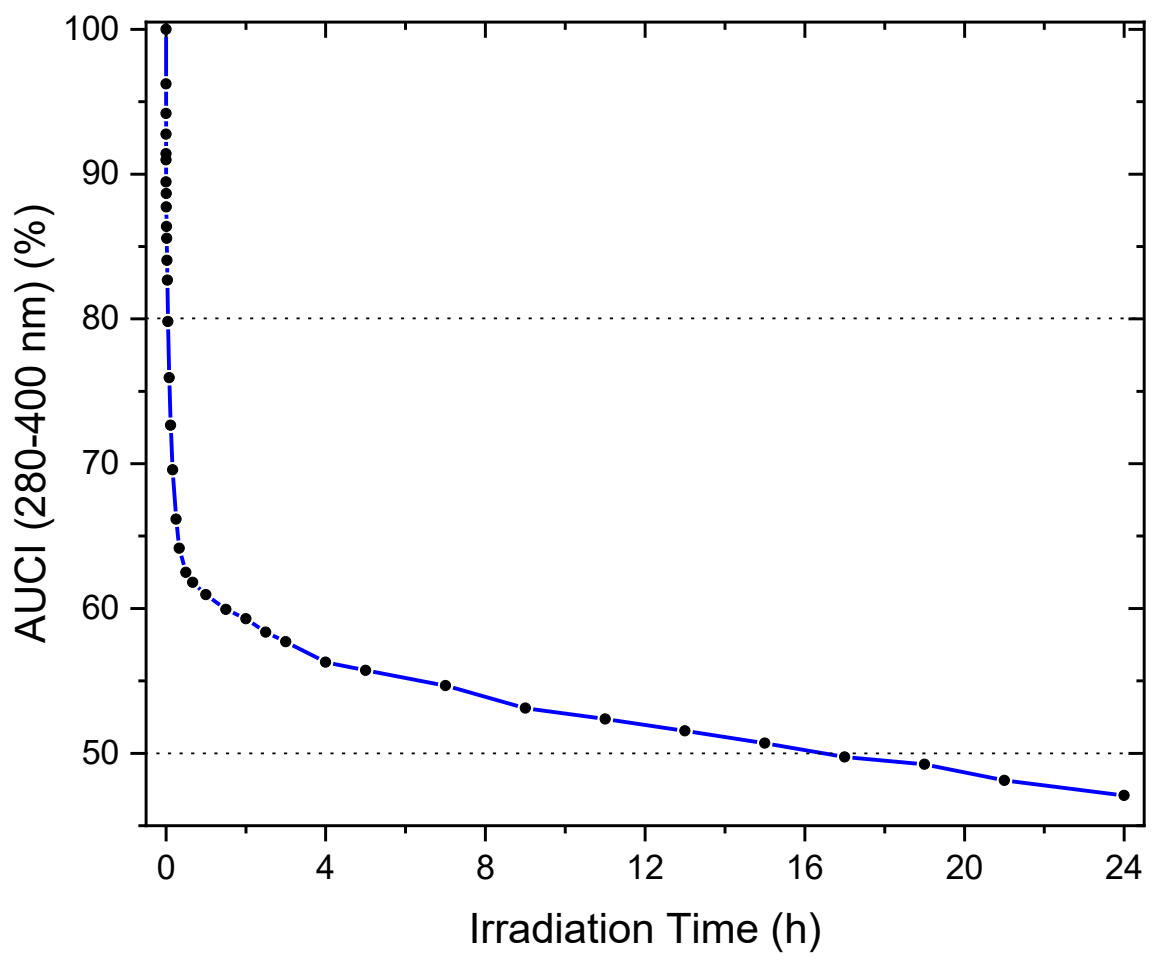

Figure S8 Area under the curve index (AUCI = 100% × AUC post-irradiation/AUC pre-irradiation; where AUC is short for the area under the curve) of the absorption spectra in Figure 1 of the main manuscript between 280 and 400 nm at the various solar simulator irradiation times. A dotted line is used to mark the point of both an AUCI of 80% (0.05 h irradiation) and 50% (16.5 h irradiation).

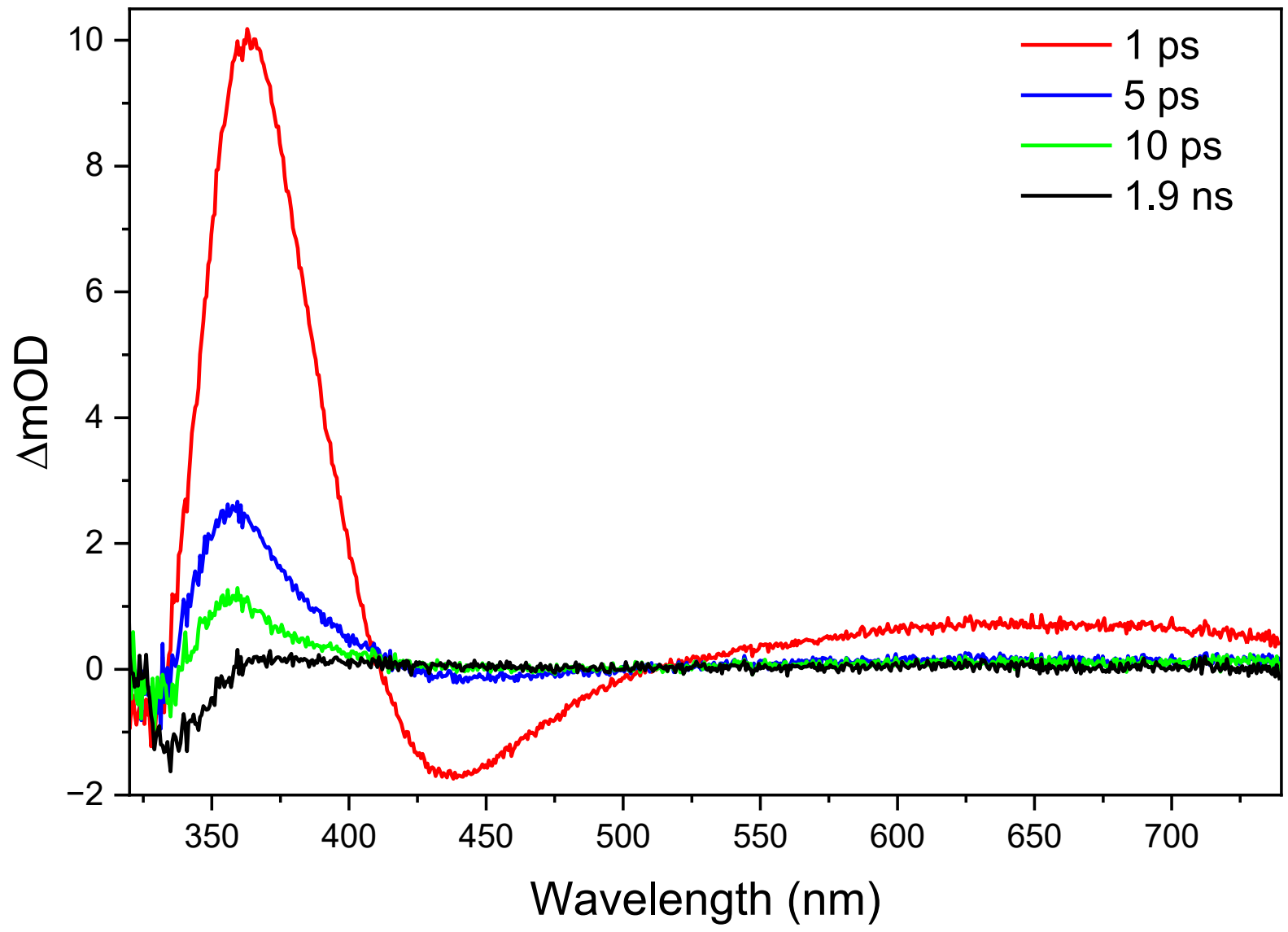

Figure S9 Transient electronic absorption spectra at select pump-probe time delays for **3pCA** at 65 µM in methanol following photoexcitation at 315 nm.

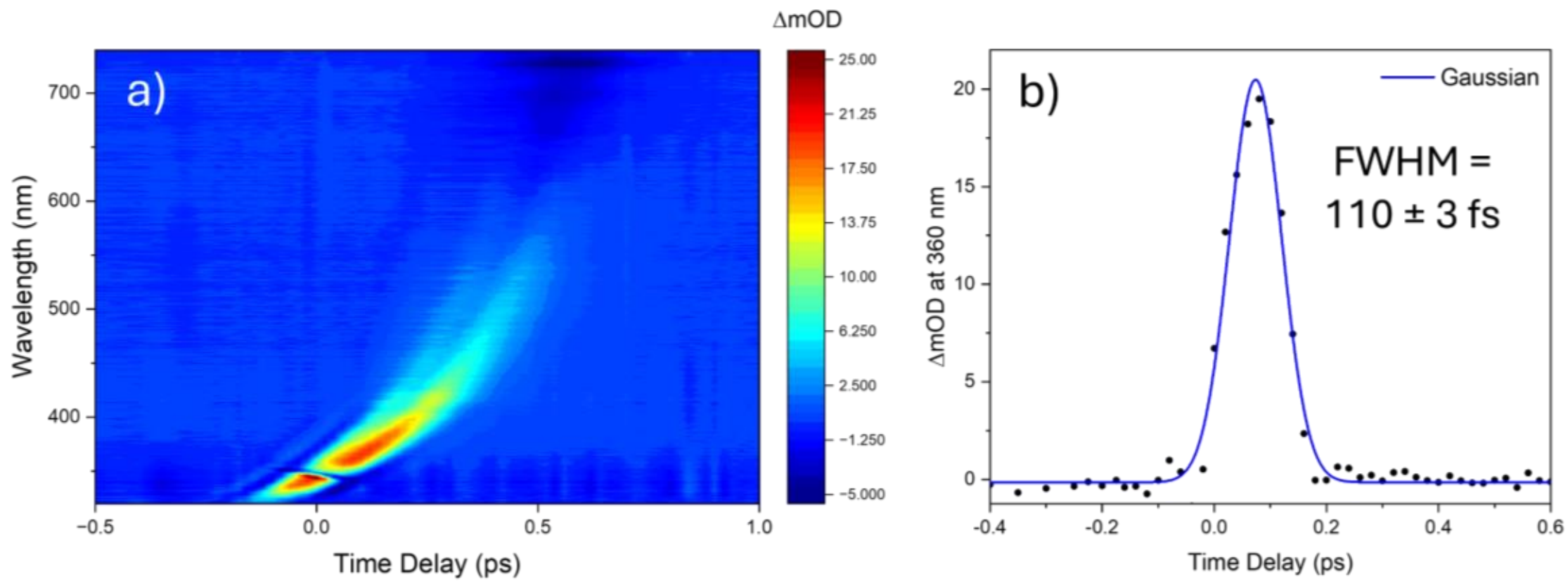

Figure S10 a) Transient electronic absorption spectra of methanol at a pump wavelength of 315 nm, presented as a false colour heatmap. b) Integrated transient slice from a) at a probe wavelength of 360 nm fit with a Gaussian (blue line) to estimate the instrument response of the transient absorption experiment. The full width half maximum (FWHM) of the Gaussian is 110 ± 3 fs, where the error pertains to one standard error between the fit and the raw data.

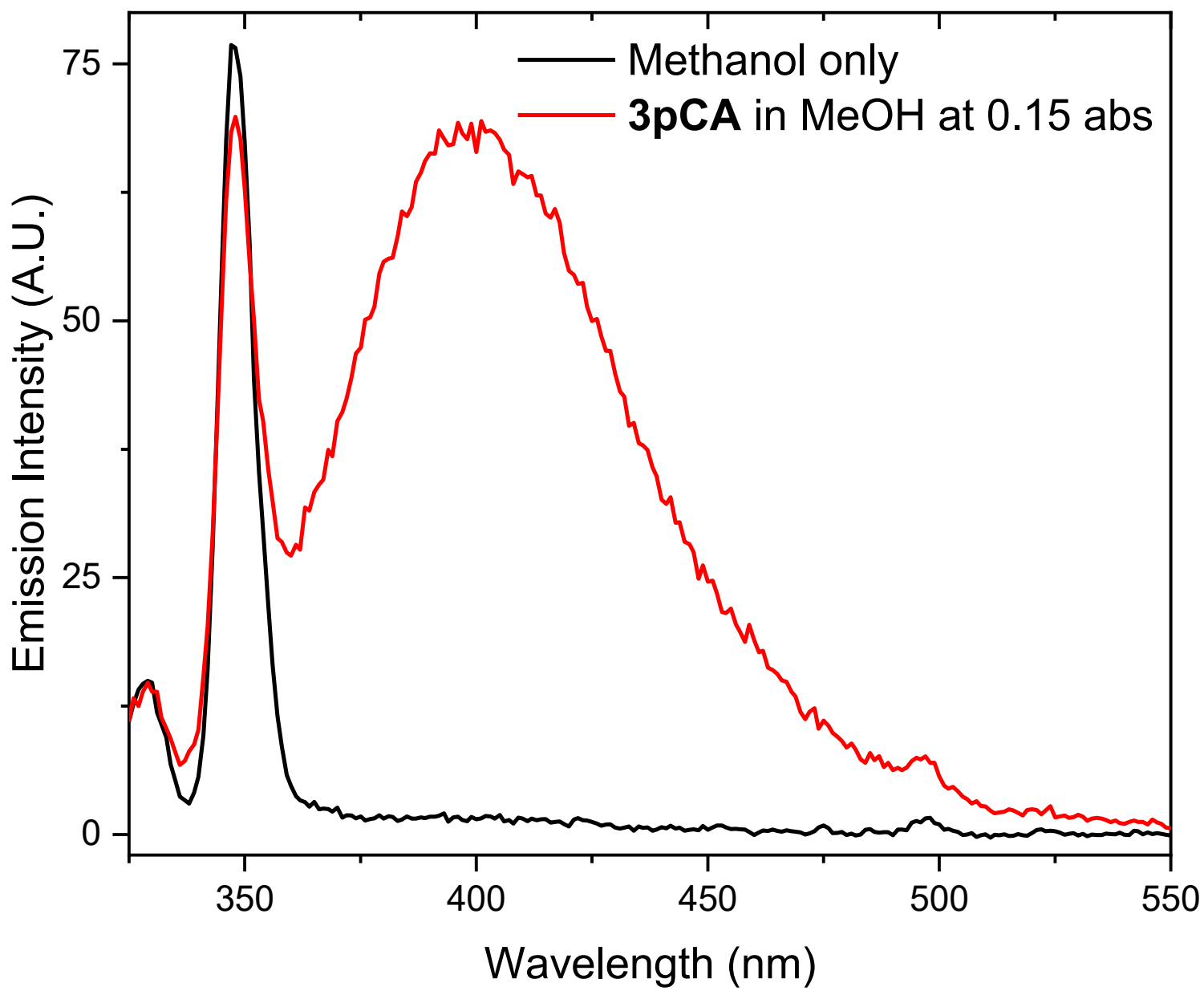

Figure S11 Emission spectrum of **3pCA** in methanol (red line; absorbance of 0.15 in a 1 cm cuvette) and methanol (black line) as a background to highlight the Raman scatter peak. The emission quantum yield was found to be negligible (<1%; data not included), which can also be inferred here from the relative intensity to the Raman scatter.

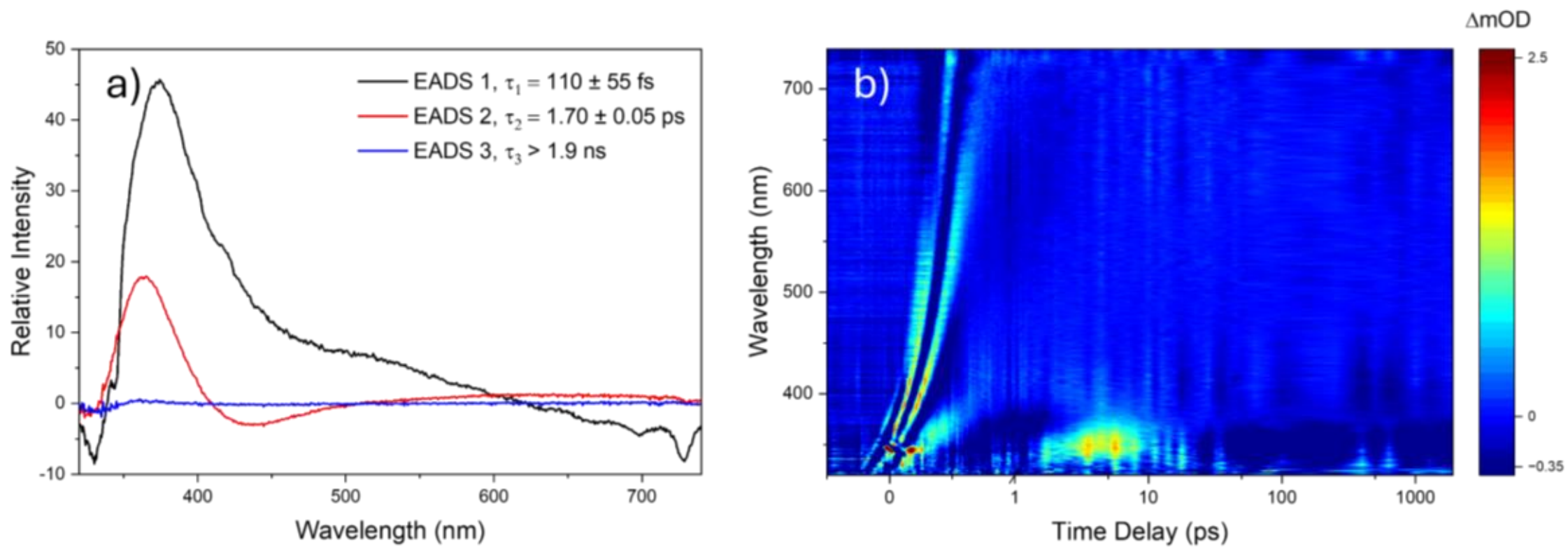

Figure S12 a) Evolution associated difference spectra (EADS) obtained from the global sequential fitting model in the Glotaran software package for the transient electronic absorption data presented in Figure 2 of the main text. The corresponding lifetimes for the EADS are presented in a). b) Fitting residuals presented as a false colour heatmap (ΔmOD limits set to 10% of the limits in Figure 2 of the main text to aid in assessing the quality of the fit).

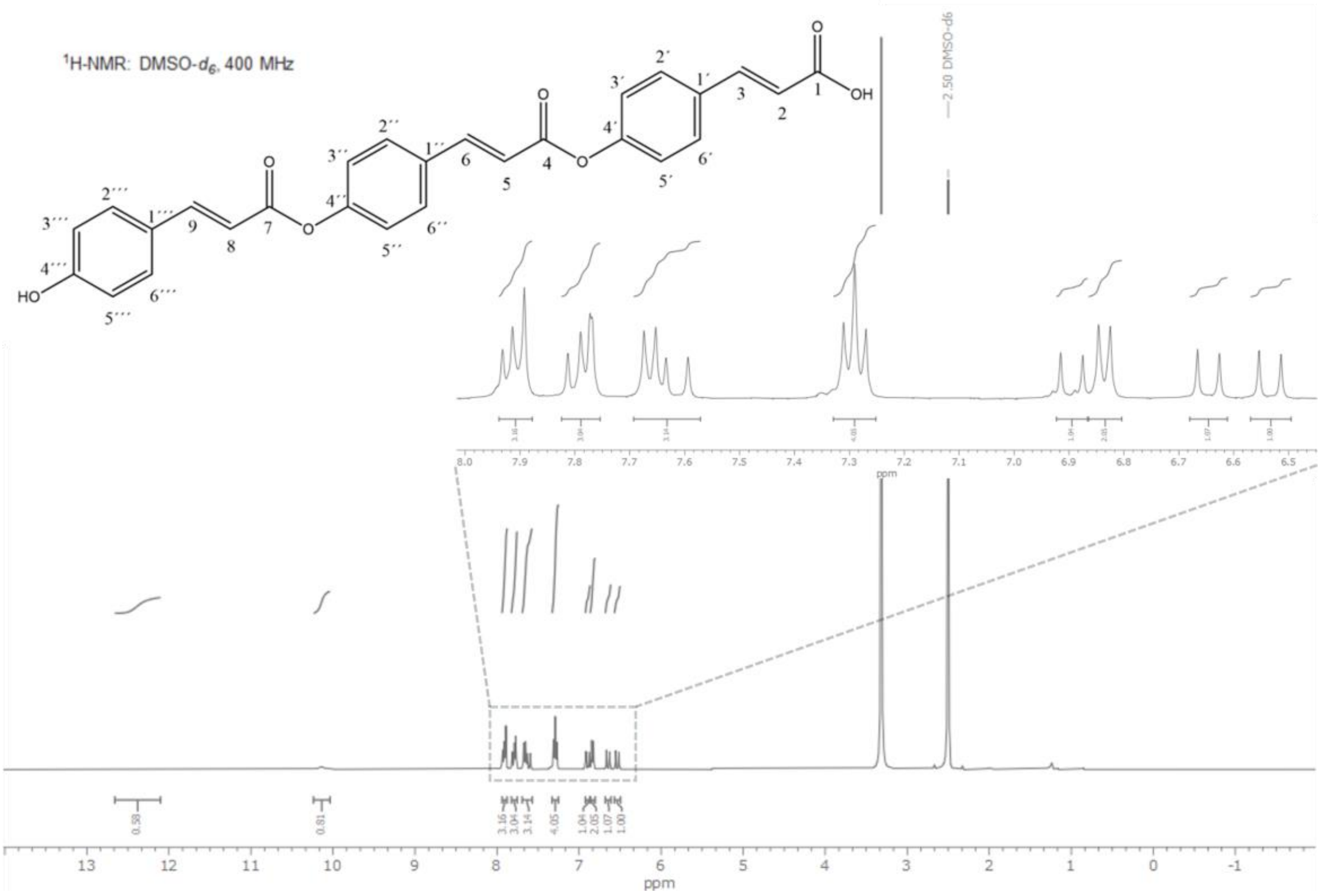

Figure S13 ^{1}H-NMR of **3pCA** in DMSO-d_6 to confirm all *trans* form pre-irradiation (to note, irradiation measurements in DMSO are not suitable due to unwanted photochemistry). ^{1}H-NMR (400 MHz, DMSO-d_6): δ 12.40 (bs, 1H, -COOH), 10.14 (bs, 1H, -OH), 7.92 (d, 1H, J = 16.0 Hz, H9), 7.90 (d, 2H, J = 8.4 Hz, H2´6´), 7.79 (d, 1H, J = 16.0 Hz, H6), 7.78 (d, 2H, J = 8.3 Hz, H2´´6´´), 7.66 (d, 2H, J = 8.5 Hz, H2´´´6´´´), 7.61 (d, 1H, J = 16.0 Hz, H3), 7.30 (d, 2H, J = 8.4 Hz, H3´5´), 7.28 (d, 2H, J = 8.3 Hz, H3´´5´´), 6.89 (d, 1H, J = 16.0 Hz, H8), 6.86 (d, 1H, J = 16.0 Hz, H5), 6.83 (d, 2H, J = 8.5 Hz, H3´´´5´´´), 6.65 (d, 1H, J = 16.0 Hz, H5), 6.53 (d, 1H, J = 16.0 Hz, H2).
